# Tezepelumab in patients with asthma: a systematic review and meta-analysis of randomized controlled trials

**DOI:** 10.1016/j.clinsp.2026.101028

**Published:** 2026-06-30

**Authors:** Wenli Shang, Guizuo Wang, Dong Han

**Affiliations:** Department of Respiratory and Critical Care Medicine, Shaanxi Provincial People’s Hospital, Xi’an, Shaanxi, China

**Keywords:** Tezepelumab, Asthma, Efficacy, Meta-analysis

## Abstract

**Objective:**

Current biological treatments are not suitable for many patients with severe asthma. The aim of this meta-analysis was to determine the efficacy of tezepelumab in patients with asthma.

**Methods:**

The authors conducted systematic searches of PubMed, Embase, the Cochrane Library, and clinicaltrials.gov without language restrictions. Randomized Controlled Trials (RCTs) on treatment of asthma with tezepelumab were reviewed. The authors summarized findings as Risk Ratios (RR) for dichotomous outcomes and Weighted Mean Differences (WMD) for continuous outcomes, each with 95% Confidence Intervals (95% CI).

**Results:**

Seven RCTs (enrolling 2050 participants) met the inclusion criteria. Tezepelumab showed significant effects on asthma exacerbation (RR = 0.72, 95% CI 0.65% to 0.80), percentage change in Forced Expiratory Volume in 1 s (FEV_1_) (WMD = 7.97%, 95% CI 3.10% to 13.83%), percentage change in Forced Vital Capacity (FVC) (WMD = 3.80%, 95% CI 2.48% to 5.12%), Asthma Control Questionnaire (ACQ)-6 score (WMD = -0.35, 95% CI -0.45 to -0.25), Asthma Quality of Life Questionnaire (standardized) for patients 12-years of age or older (AQLQ[S]+12) score (WMD = 0.33, 95% CI 0.22 to 0.43), and serious adverse events (RR = 0.74, 95% CI 0.57 to 0.95).

**Conclusions:**

Tezepelumab appears safe and effective; further long-term and real-world studies are warranted.

## Highlights

• Tezepelumab significantly improved exacerbation, FEV1, FVC, ACQ-6 and AQLQ(S)+12.
• Tezepelumab was safe in patients with asthma.
• Tezepelumab should be considered in asthmatic patients, especially those with severe asthma.

## Introduction

Approximately 3%‒10% of patients with asthma have severe asthma, with poorly controlled symptoms and recurrent exacerbations, despite receiving optimized standard-of-care treatment.^1^ Monoclonal antibodies targeting specific immune pathways, such as Immunoglobulin (Ig) E or Type 2 (T2) cytokines (interleukins-4, −5, and −13) and their receptors, improve disease control in some patients with severe asthma.^1^ However, current biological treatments are not suitable for many patients with severe asthma, especially those with a non-allergic or non-eosinophilic phenotype.^2^^,^^3^ Thymic Stromal Lymphopoietin (TSLP) is an epithelial cell-derived cytokine involved in multiple downstream pathways in asthma pathophysiology.^4^^,^^5^ The expression of TSLP is increased in the airways of asthmatics, and its levels are associated with T2 cytokine and chemokine expression, disease severity, airway obstruction, and glucocorticoid resistance.^6^^,^^7^ Tezepelumab is a human monoclonal antibody (IgG2λ) that specifically binds to TSLP and blocks its interaction with its receptor complex.^8^ Only a few Randomized Controlled Trials (RCTs) have evaluated the efficacy and safety of tezepelumab in patients with asthma, and their results differed.

The previous meta-analysis^9^ found that long-term (12‒52 weeks) use of tezepelumab in patients with asthma does not increase the incidence of adverse events. Therefore, the aim of this study was to conduct a systematic review and meta-analysis of RCTs to clarify the efficacy profile of tezepelumab in asthma.

## Methods

### Data sources and search strategy

This meta-analysis was based on the Preferred Reporting Items for Systematic Reviews and Meta-Analyses (PRISMA) statement.^10^ The protocol was previously registered in July 2022 in the PROSPERO database (Review register: CRD42022345592), link https://www.crd.york.ac.uk/PROSPERO/view/CRD42022345592. The PubMed, Embase, Cochrane Library, and clinicaltrials.gov were searched for studies up to May 2025.

### Study selection

To be eligible for inclusion in this meta-analysis, studies had to meet the following criteria: a) Inclusion of asthma patients according to Global Initiative for Asthma (GINA) 2021;[1] b) Use of a randomized controlled design to make a comparison of tezepelumab with placebo or blank; and c) Follow-up for 12-weeks or longer to observe the efficacy and safety. The search strings used for the databases were (“tezepelumab” OR “Tezspire” OR “AMG157” OR “MEDI9929”) AND “asthma”. In addition, the authors screened the reference lists of relevant review articles to identify studies that might have been missed. There were no language restrictions on the study screening process.

### Data extraction and quality assessment

Two reviewers independently screened articles based on the inclusion criteria. Reviewers compared the selected studies and reached consensus on differences. The authors extracted the required data from the included studies, including authors, baseline characteristics, interventions, and outcomes such as asthma exacerbation, Forced Expiratory Volume in 1 s (FEV_1_), Forced Vital Capacity (FVC), Asthma Control Questionnaire (ACQ)-6 score, Asthma Quality of Life Questionnaire (standardized) for patients 12-years of age or older (AQLQ[S]+12) total score, and mortality, and adverse events and serious adverse events.

### Risk of bias of included trials

Two reviewers independently assessed the risk of bias using the Cochrane Collaboration Risk of Bias tool (RoB2) for RCTs.^11^ Five domains of bias (i.e., randomization process, deviations from intended interventions, missing outcome data, measurement of the outcome, and selection of the reported results) were evaluated and reported. Disagreements were resolved by discussion and consensus.

### Data synthesis and statistical analysis

Data were analyzed using RevMan Version 5.1 (The Cochrane Collaboration). The authors calculated Risk Ratios (RR) for dichotomous variables and Weighted Mean Differences (WMD) for continuous variables with corresponding 95% Confidence Intervals (95% CI). Heterogeneity was assessed using the chi-squared-based Q statistic method and Higgins I2 tests. If there was significant heterogeneity (Chi-Squared test p ≤ 0.10 or I2 ≥ 50%), a random effects model was used, a fixed effects model was used. Subgroup analyses were performed according to the baseline blood eosinophil count; p-values <0.05 indicate statistical significance. The p-value of Egger’s linear regression test^12^ and Begg’s rank correlation test^13^^,^^14^ (STATA version 12.0) were used to assess the presence of publication bias.

To assess the robustness of the results, sensitivity analyses were performed in the following ways: changing the effect model, excluding studies using tezepelumab intravenously, excluding studies with follow-up < 26-weeks, excluding trials including patients with non-severe asthma, or excluding trials including patients < 18-years of age.

## Results

### Study selection and characteristics

**Fig. 1:**
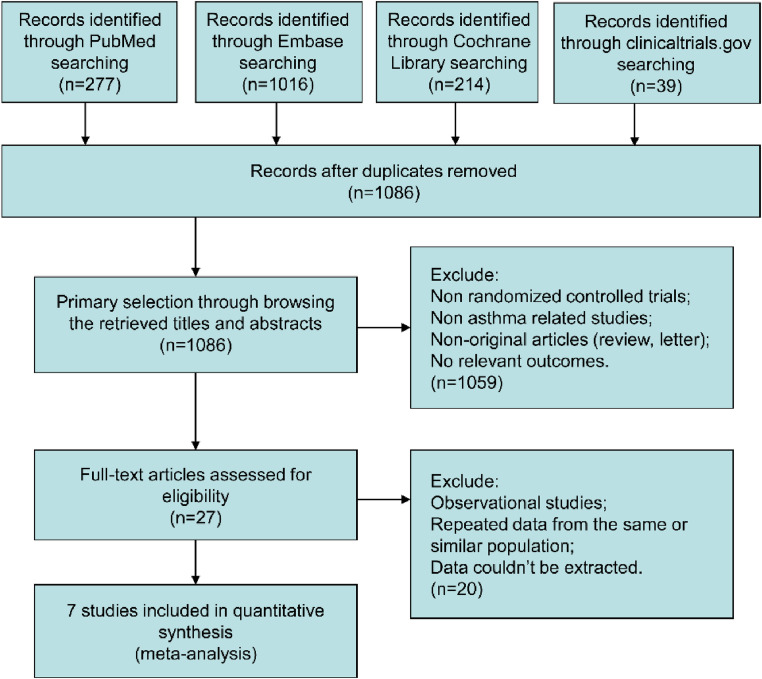
Flow chart for selection of studies.

A total of 1546 trials were initially searched, of which 7 studies^8^^,^^15^, ^16^, ^17^, ^18^, ^19^, ^20^ were included in the meta-analysis (Fig. 1). Table 1 lists the baseline characteristics of these trials. There were 1168 patients in the tezepelumab group and 882 patients in the control group. The risk of bias results are summarized in Figure S1.

**Table 1:** Baseline characteristics of trials included in meta-analysis.

Study (Ref.)^a^	Year	Follow-up weeks	Regimen	n	Age, years (SD)	Male, %	BMI, kg/m^2^ (SD)	Blood eosinophil count, cells/μL (SD)	FeNO, ppb (SD)
Cole VECTOR^15^	2023	28	210 mg every 4-weeks	35	16.3 (2.3)	60	23.3 (4.7)	NR	NR
			Placebo^a^	35	16.6 (3.1)	69	28.0 (7.0)	NR	NR
Corren PATHWAY^16^	2017	52	70 mg every 4-weeks	14	50.6 (12.4)	35	28.3 (5.1)	345 (284)	34.5 (46.9)
			210 mg every 4-weeks	145	52.6 (12.5)	37	28.4 (4.9)	359 (347)	30.4 (29.4)
			280 mg every 2-weeks	146	50.1 (12.2)	36	27.7 (5.0)	378 (423)	32.6 (33.9)
			Placebo^a^	148	52.2 (11.5)	32	28.5 (5.5)	366 (323)	36.3 (38.9)
Diver CASCADE^17^	2021	28	210 mg every 4-weeks	59	50.4 (12.7)	34	30.6 (5.8)	302 (307)	33.0 (39.4)
			Placebo^a^	57	50.4 (13.9)	54	28.4 (6.4)	272 (161)	31.2 (19.9)
Gauvreau^8^	2014	12	700 mg every 4-weeks	16	30.8 (2.7)	38	24.9 (0.7)	296.5 (40.2)	42.3 (4.3)
			Placebo^b^	15	31.5 (2.9)	27	26.5 (1.1)	281.1 (57.2)	58.9 (14.3)
Menzies-Gow NAVIGATOR^18^	2021	52	210 mg every 4-weeks	528	49.9 (16.3)	37	28.7 (7.1)	327 (293)	41.4 (36.3)
			Placebo^a^	531	49.0 (15.9)	37	28.3 (6.9)	353 (488)	46.3 (44.7)
Sverrild UPSTREAM^19^	2021	12	700 mg every 4 weeks	20	42 (20)	45	26.5 (4.3)	266 (177)	39.8 (36.1)
			Placebo^b^	20	40 (15)	40	29.0 (5.2)	280 (203)	37.0 (30.0)
Wechsler SOURCE^20^	2022	48	210 mg every 4 weeks	74	53.5 (12.1)	34	29.3 (6.7)	253 (203)	38.7 (40.8)
			Placebo^a^	76	53.4 (11.9)	41	29.4 (7.4)	232 (154)	42.4 (37.4)

BMI, Body Mass Index; FeNO, Fraction of exhaled Nitric Oxide; NR, Not Reported; ppb, Parts per billion; SD, Standard Deviation.

a Subcutaneously

b Intravenously.

### Asthma exacerbation

**Fig. 2:**
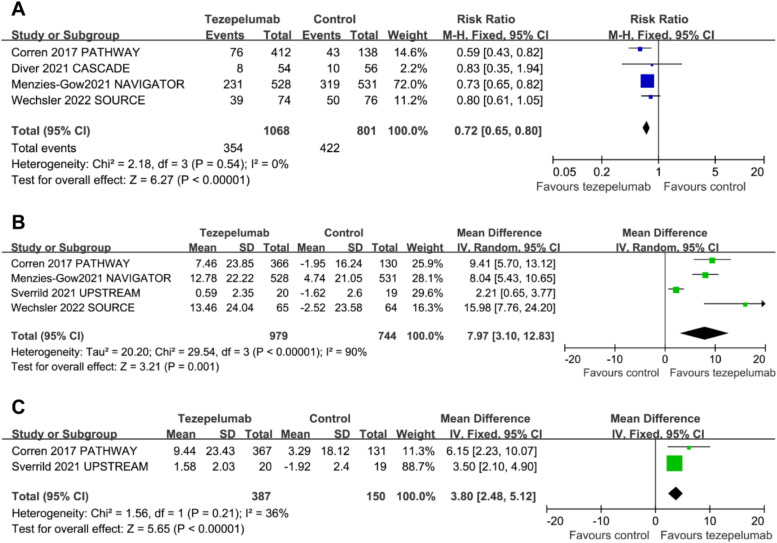
Compared with the control groups, tezepelumab showed significant effects on (A) Asthma exacerbation (RR = 0.72, 95% CI 0.65 to 0.80), (B) Percentage change in FEV_1_ (WMD = 7.97%, 95% CI 3.10% to 13.83%), and (C) Percentage change in FVC (WMD = 3.80%, 95% CI 2.48% to 5.12%).

Data on patients with at least one asthma exacerbation were available from four RCTs (including 1869 patients). Compared with the control conditions, the percentage of patients with at least one asthma exacerbation was significantly lower in the tezepelumab group (RR = 0.72, 95% CI 0.65 to 0.80; p < 0.00001 [Fig. 2A]). There was no significant heterogeneity (I2 = 0%; p = 0.54). The proportion of patients with exacerbations was 33.1% in the tezepelumab group versus 52.7% in controls. Egger’s test (p = 0.193) and Begg’s test (p = 0.089) did not show evidence of publication bias.

### Forced expiratory volume in 1 second

Four trials reported data on the percentage change in FEV_1_ from baseline (1723 patients). Tezepelumab significantly increased FEV_1_ compared to the control group (WMD = 7.97%, 95% CI 3.10% to 13.83%; p = 0.001 [Fig. 2B]). There was significant heterogeneity (I2 = 90%; p < 0.00001). Egger’s test (p = 0.201) and Begg’s test (p = 0.174) did not show evidence of publication bias.

### Forced vital capacity

The percentage change in FVC from baseline was evaluated in two RCTs (537-patients). Tezepelumab significantly improved FVC (WMD = 3.80%, 95% CI 2.48% to 5.12%; p < 0.00001 [Fig. 2C]). There was no significant heterogeneity (I2 = 36%; p = 0.21).

### ACQ-6 score

**Fig. 3:**
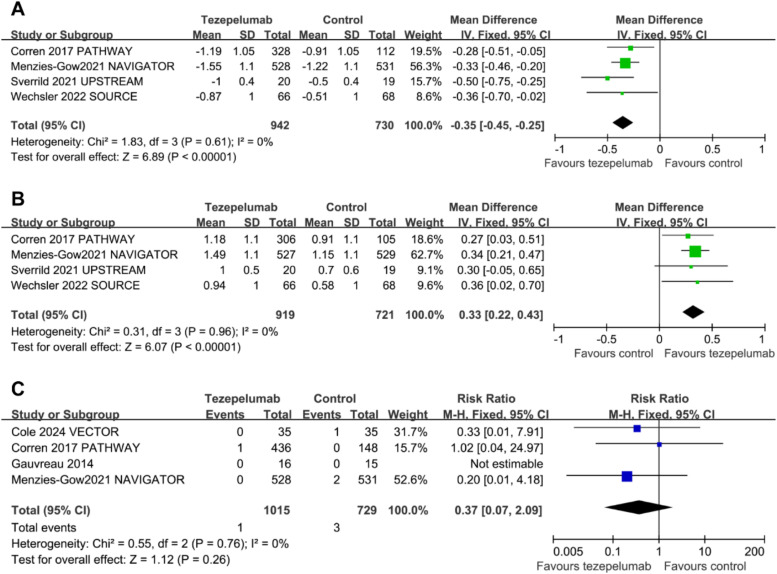
Forest plot assessing the efficacy of tezepelumab on (A) Asthma Control Questionnaire-6 score (WMD = −0.35, 95% CI −0.45 to −0.25), (B) Asthma Quality of Life Questionnaire (standardized) for patients 12-years of age or older total score (WMD = 0.33, 95% CI 0.22 to 0.43), and (C) Mortality (RR = 0.37, 95% CI 0.07 to 2.09).

Data on the change of the ACQ-6 score were available from four RCTs (1672 patients). Tezepelumab significantly improved the ACQ-6 score (WMD = −0.35, 95% CI −0.45 to −0.25; p < 0.00001 [Fig. 3A]). There was no significant heterogeneity (I2 = 0%; p = 0.61). Egger’s test (p = 0.224) and Begg’s test (p = 0.308) did not show evidence of publication bias.

### AQLQ(S)+12 score

Four RCTs reported data on the change of AQLQ(S)+12 score (1640 patients). Tezepelumab significantly improved AQLQ(S)+12 score (WMD = 0.33, 95% CI 0.22 to 0.43; p < 0.00001 [Fig. 3B]). There was no significant heterogeneity (I2 = 0%; p = 0.96). Egger’s test (p = 0.431) and Begg’s test (p = 0.308) did not show evidence of publication bias.

### Mortality

Four RCTs reported data on mortality (1744-patients), and only three trials had patient deaths. There was no statistically significant difference in all-cause mortality between the two groups (RR = 0.37, 95% CI 0.07 to 2.09; p = 0.26 [Fig. 3C]). There was no significant heterogeneity (I2 = 0%; p = 0.76). The mortality in the tezepelumab group was 0.10% compared with 0.41% in the control group. Egger’s test (p = 0.361) and Begg’s test (p = 0.296) did not show evidence of publication bias.

### Adverse events

**Fig. 4:**
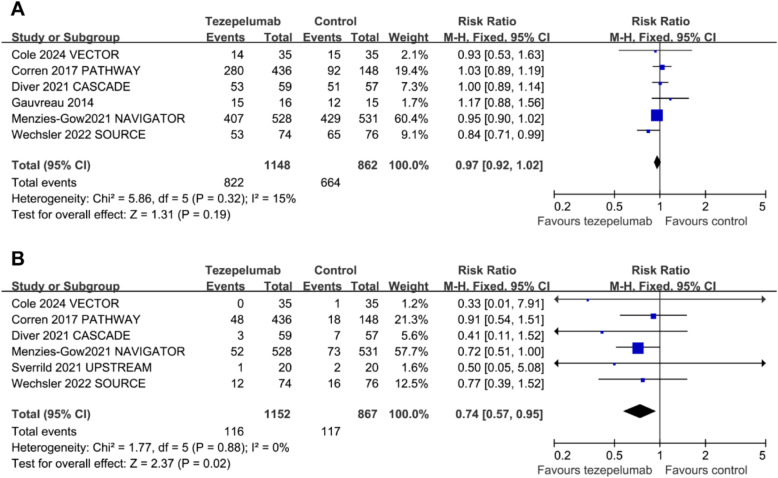
Forest plot assessing the safety of tezepelumab, (A) Adverse events (RR = 0.97, 95% CI 0.92 to 1.02), and (B) Serious adverse events (RR = 0.74, 95% CI 0.57 to 0.95).

Adverse events were reported in six RCTs (2010-patients). The number of patients with at least one adverse event did not differ significantly between the two groups (RR = 0.97, 95% CI 0.92 to 1.02; p = 0.19 [Fig. 4A]), with a rate of 71.60% versus 77.03%. There was no significant heterogeneity (I2 = 15%; p = 0.32). Egger’s test (p = 0.670) and Begg’s test (p = 0.707) did not show evidence of publication bias.

### Serious adverse events

Data on serious adverse events were extracted from six studies (2019-patients). The number of patients with at least one serious adverse event was significantly lower in the tezepelumab group (RR = 0.74, 95% CI 0.57 to 0.95; p = 0.02 [Fig. 4B]), with a rate of 10.07% versus 13.49%. There was no significant heterogeneity (I2 = 0%; p = 0.88). Egger’s test (p = 0.237) and Begg’s test (p = 0.260) did not show evidence of publication bias.

### Subgroup analysis

Since the blood eosinophil count may influence the efficacy of tezepelumab, the authors probed into detailed results in subgroup analyses stratified by baseline blood eosinophil count (≥ 300 cells/μL or < 300 cells/μL). The results are summarized in Table 2.

**Table 2:** Subgroup analysis of the meta-analysis.

Outcomes	Baseline blood eosinophil count	Number of trials	Effect (95% CI)	Estimate for overall effect	Heterogeneity
Asthma exacerbation	≥ 300 cells/μL	2	0.71 (0.63 to 0.79)	p < 0.00001	*I^2^* = 30%, p = 0.23
	< 300 cells/μL	2	0.81 (0.62 to 1.05)	p = 0.11	*I^2^* = 0%, p = 0.94
FEV_1_	≥ 300 cells/μL	2	8.49 (6.36 to 10.63)	p < 0.00001	*I^2^* = 0%, p = 0.55
	< 300 cells/μL	2	8.48 (−4.96 to 21.92)	p = 0.22	*I^2^* = 90%, p = 0.001
Forced vital capacity	≥ 300 cells/μL	1	6.15 (2.23 to 10.07)	p = 0.002	Not applicable
	< 300 cells/μL	1	3.50 (2.10 to 4.90)	p < 0.00001	Not applicable
ACQ-6 score	≥ 300 cells/μL	2	−0.32 (−0.43 to −0.20)	p < 0.00001	*I^2^* = 0%, p = 0.71
	< 300 cells/μL	2	−0.45 (−0.65 to −0.25)	p < 0.0001	*I^2^* = 0%, p = 0.52
AQLQ(S)+12 score	≥ 300 cells/μL	2	0.32 (0.21, 0.44)	p < 0.00001	*I^2^* = 0%, p = 0.62
	< 300 cells/μL	2	0.33 (0.09, 0.57)	p = 0.008	*I^2^* = 0%, p = 0.81
Mortality	≥ 300 cells/μL	2	0.39 (0.05 to 3.05)	p = 0.37	*I^2^* = 0%, p = 0.47
	< 300 cells/μL	1	Not estimable	Not applicable	Not applicable
Adverse events	≥ 300 cells/μL	2	0.97 (0.92 to 1.03)	p = 0.37	I^2^ = 5%, p = 0.30
	< 300 cells/μL	3	0.97 (0.82 to 1.15)	p = 0.76	*I^2^* = 62%, p = 0.07
Serious adverse events	≥ 300 cells/μL	2	0.77 (0.58 to 1.01)	p = 0.06	*I^2^* = 0%, p = 0.45
	< 300 cells/μL	3	0.65 (0.36 to 1.15)	p = 0.14	*I^2^* = 0%, p = 0.69

ACQ, Asthma Control Questionnaire; AQLQ(S)+12, Asthma Quality of Life Questionnaire (standardized) for patients 12-years of age or older; CI, Confidence Interval; FEV_1_, Forced Expiratory Volume in 1 s.

## Sensitivity analysis

The presemt results were mostly confirmed in sensitivity analyses. A significant change was only observed in the outcome for serious adverse events when excluding trials including patients < 18-years of age. In this analysis, no significant changes were found in outcomes for asthma exacerbation, FEV1, FVC, ACQ-6 score, AQLQ(S)+12 score, mortality, or adverse events (Table 3).

**Table 3:** Results of sensitivity analyses.

	Number of trials	Heterogeneity		RR or WMD (95% CI)	p-value
		p-value	*I^2^*		
Changing the effect model					
Asthma exacerbation	4	0.54	0%	0.72 (0.65, 0.80)^R^	<0.00001
FEV_1_	4	<0.00001	90%	4.66 (3.42, 5.90)^F^	<0.00001
Forced vital capacity	2	0.21	36%	4.17 (1.91, 6.42)^R^	0.0003
ACQ-6 score	4	0.61	0%	−0.35 (−0.45, −0.25)^R^	<0.00001
AQLQ(S)+12 score	4	0.96	0%	0.33 (0.22, 0.43)^R^	<0.00001
Mortality	3	0.76	0%	0.40 (0.07, 2.43)^R^	0.32
Adverse events	6	0.32	15%	0.97 (0.91, 1.03)^R^	0.31
Serious adverse events	6	0.88	0%	0.74 (0.58, 0.96)^R^	0.02
Excluding studies using tezepelumab intravenously					
FEV_1_	3	0.19	40%	8.97 (6.90, 11.03)^F^	<0.00001
Forced vital capacity	1	NA	NA	6.15 (2.23, 10.07)^F^	0.002
ACQ-6 score	3	0.91	0%	−0.32 (−0.43, −0.21)^F^	<0.00001
AQLQ(S)+12 score	3	0.87	0%	0.33 (0.22, 0.44)^F^	<0.00001
Mortality	3	0.76	0%	0.37 (0.07, 2.09)^F^	0.26
Adverse events	5	0.40	0%	0.96 (0.91, 1.01)^F^	0.15
Serious adverse events	5	0.80	0%	0.74 (0.58, 0.95)^F^	0.02
Excluding studies follow-up < 26-weeks					
FEV_1_	3	0.19	40%	8.97 (6.90, 11.03)^F^	<0.00001
Forced vital capacity	1	NA	NA	6.15 (2.23, 10.07)^F^	0.002
ACQ-6 score	3	0.91	0%	−0.32 (−0.43, −0.21)^F^	<0.00001
AQLQ(S)+12 score	3	0.87	0%	0.33 (0.22, 0.44)^F^	<0.00001
Mortality	3	0.76	0%	0.37 (0.07, 2.09)^F^	0.26
Adverse events	5	0.40	0%	0.96 (0.91, 1.01)^F^	0.15
Serious adverse events	5	0.80	0%	0.74 (0.58, 0.95)^F^	0.02
Excluding trials including patients with non-severe asthma					
Mortality	2	0.47	0%	0.39 (0.05, 3.05)^F^	0.37
Adverse events	4	0.26	25%	0.96 (0.91, 1.01)^F^	0.16
Serious adverse events	5	0.82	0%	0.74 (0.58, 0.96)^F^	0.02
Excluding trials including patients < 18-years of age					
Asthma exacerbation	3	0.33	9%	0.69 (0.56, 0.85)^F^	0.0005
FEV_1_	3	<0.0001	91%	8.33 (1.20, 15.45)^R^	0.02
ACQ-6 score	3	0.44	0%	−0.37 (−0.52, −0.22)^F^	<0.00001
AQLQ(S)+12 score	3	0.91	0%	0.30 (0.13, 0.47)^F^	0.0006
Mortality	2	NA	NA	1.02 (0.04, 24.97)^F^	0.99
Adverse events	4	0.14	45%	0.99 (0.90, 1.08)^F^	0.77
Serious adverse events	4	0.71	0%	0.78 (0.53, 1.14)^F^	0.20

ACQ, Asthma Control Questionnaire; AQLQ(S)+12, Asthma Quality of Life Questionnaire (standardized) for patients 12-years of age or older; CI, Confidence Interval; F, Fixed-effect model; FEV_1_, Forced Expiratory Volume in 1 s; NA, Not Applicable; R, Random-effect model; RR, Risk Ratio; WMD, Weighted Mean Difference.

## Discussion

The aim of this systematic review and meta-analysis was to assess the efficacy of tezepelumab in asthma. Based on the current results, the authors found that tezepelumab significantly improved asthma exacerbation, FEV1, FVC, ACQ-6 and AQLQ(S)+12 scores. Subgroup analysis showed that tezepelumab failed to improve asthma exacerbation or FEV1 in the subgroup with low blood eosinophil count (< 300 cells/μL). This may indicate that tezepelumab is more effective in patients with high blood eosinophil counts. High heterogeneity was observed in the percentage change in FEV_1_, which was mainly attributed to the Sverrild study.^19^ This may be related to the study’s small sample size, short trial duration, and different administration routes. Unlike the safety outcomes (adverse events leading to discontinuation of study treatment, upper respiratory tract infection, influenza, bronchitis, nasopharyngitis, headache, and hypertension) in the previous meta-analysis,^9^ the focus of the present meta-analysis is on the efficacy outcomes.

TSLP is an epithelial cell-derived cytokine that activates and promotes airway inflammation and has been implicated in multiple cell types and inflammatory pathways in both T2- and non-T2-mediated asthma pathophysiology.^21^^,^^22^ Inhalational epithelial injury, including allergic and non-allergic factors such as diesel particles, cigarette smoke, viruses and bacteria, triggers the release of TSLP from epithelial cells, which activate and promote the inflammatory response in asthma.^23^, ^24^, ^25^ TSLP activates multiple immune cells that promote T2 inflammation by upregulating T2 cytokines, such as dendritic cells, group 2 innate lymphocytes, and mast cells.^22^ A variety of inflammatory cells are also activated by TSLP in non-T2 immunity, including mast cells, natural killer T-cells, innate lymphocytes, basophils, and neutrophils.^16^^,^^25^^,^^26^ Furthermore, TSLP promotes asthmatic airway remodeling by increasing fibroblast collagen production and airway smooth muscle proliferation.^27^ The EGEA study^28^ investigated plasma TSLP levels in adults with non-severe asthma. High TSLP levels were associated with the persistence of asthma attacks, poor lung function, and dyspnea after 10-years. TSLP could serve as a predictive marker for asthma persistence in patients with non-severe asthma.

A previous Bayesian network meta-analysis^29^ conducted an indirect comparison of tezepelumab, dupilumab, benralizumab, and mepolizumab in the treatment of eosinophilic asthma. Tezepelumab was associated with significantly lower exacerbation rates than benralizumab and larger improvements in FEV_1_ compared to mepolizumab and benralizumab. Another study^30^ compared the efficacy of tezepelumab with other approved biologics (dupilumab, benralizumab, mepolizumab, reslizumab, and omalizumab) via indirect treatment comparisons in patients aged ≥12-years with severe uncontrolled asthma. Tezepelumab was favorably associated with numerically lower annualized asthma exacerbation rate and ranked first in the network for both annualized asthma exacerbation rate and hospitalization. Habash et al.^31^ assessed the cost-effectiveness of tezepelumab as an add-on maintenance therapy, compared with the Standard of Care (SoC), for the treatment of patients with severe asthma in Canada. The base case analysis suggested that tezepelumab plus SoC was associated with a Quality-Adjusted Life-Year (QALY) gain of 1.077 compared with SoC alone at an incremental cost of $207,101 (2022 Canadian dollars), resulting in an incremental cost-utility ratio of $192,357/QALY. The key scenario analysis demonstrated that tezepelumab was dominant against all currently reimbursed biologics (omalizumab, mepolizumab, benralizumab, and dupilumab), with higher incremental QALYs (ranging from 0.062 to 0.407) and lower incremental costs (ranging from -$6878 to -$1974). Additionally, when compared against currently reimbursed biologics in Canada, tezepelumab had the highest probability of being cost-effective across all Willingness-To-Pay (WTP) thresholds.

There are some limitations that need to be considered when interpreting the results of this meta-analysis. First, the sample sizes of the two included trials were small. Second, some outcomes were based on a small number of trials. Third, the dose of tezepelumab varied between studies. Fourth, potential conflicts of interest in the included industry-sponsored RCTs. Fifth, this meta-analysis was not patient-level, so the results should be considered provisional.

## Conclusions

Tezepelumab was safe and significantly improved exacerbation, FEV1, FVC, ACQ-6 and AQLQ(S)+12 scores in asthma. Tezepelumab should be considered in asthmatic patients, especially those with severe asthma.

## Data availability

Extracted data are available on request to the corresponding author.

## Ethics approval and consent to participate

Not applicable.

## Consent for publication

Not applicable.

## Authors’ contributions

Conceptualization: D.H.; Data curation: W.S. and G.W.; Formal analysis: D.H. and G.W.; Investigation: W.S. and G.W.; Methodology: W.S. and D.H.; Software: W.S. and G.W.; Supervision: D.H.; Validation: D.H. and G.W.; Writing-original draft: W.S. and G.W.; Writing-review & editing: D.H.

## Funding

This research was supported by The 10.13039/501100015401Key Research and Development Projects of Shaanxi Province (Grant n° 2023-YBSF-064). The funder had no role in the design of the study; in the collection, analyses, or interpretation of data; in the writing of the manuscript, or in the decision to publish the results.

## Declaration of competing interest

The authors declare no conflicts of interest.

## Appendix Supplementary materials

Image, application 1

Image, application 2

Image, application 3

Image, application 4
