# Supplementary material for: Tezepelumab in patients with asthma: a systematic review and meta-analysis of randomized controlled trials
Source: Clinics (Sao Paulo). 2026 Jun 30;81:101028. doi: 10.1016/j.clinsp.2026.101028 (PMC13330515; doi:10.1016/j.clinsp.2026.101028)
Supplement: Supplementary file 2 [file mmc2.docx]

**Table S1** Search strategies for all databases

**Table A** Search strategy in PubMed

| **Search strings** | **Items found** |
| --- | --- |
| (“tezepelumab” OR “Tezspire” OR “AMG157” OR “MEDI9929”) AND “asthma” | 277 |

**Table B** Search strategy in EMBASE

| **Search strings** | **Items found** |
| --- | --- |
| ('tezepelumab' OR 'tezspire' OR 'amg157' OR 'medi9929') AND 'asthma' | 1016 |

**Table C** Search strategy in Cochrane Library

| **Search strings** | **Items found** |
| --- | --- |
| (“tezepelumab” OR “Tezspire” OR “AMG157” OR “MEDI9929”) AND “asthma” | 214 |

**Table D** Search strategy in clinicaltrials.gov

| **Status** | **Condition/disease** | **Intervention/treatment** | **Items found** |
| --- | --- | --- | --- |
| All studies | “asthma” | (“tezepelumab” OR “Tezspire” OR “AMG157” OR “MEDI9929”) | 39 |
