# Supplementary material for: Tezepelumab in patients with asthma: a systematic review and meta-analysis of randomized controlled trials
Source: Clinics (Sao Paulo). 2026 Jun 30;81:101028. doi: 10.1016/j.clinsp.2026.101028 (PMC13330515; doi:10.1016/j.clinsp.2026.101028)
Supplement: Supplementary file 4 [file mmc4.pdf]

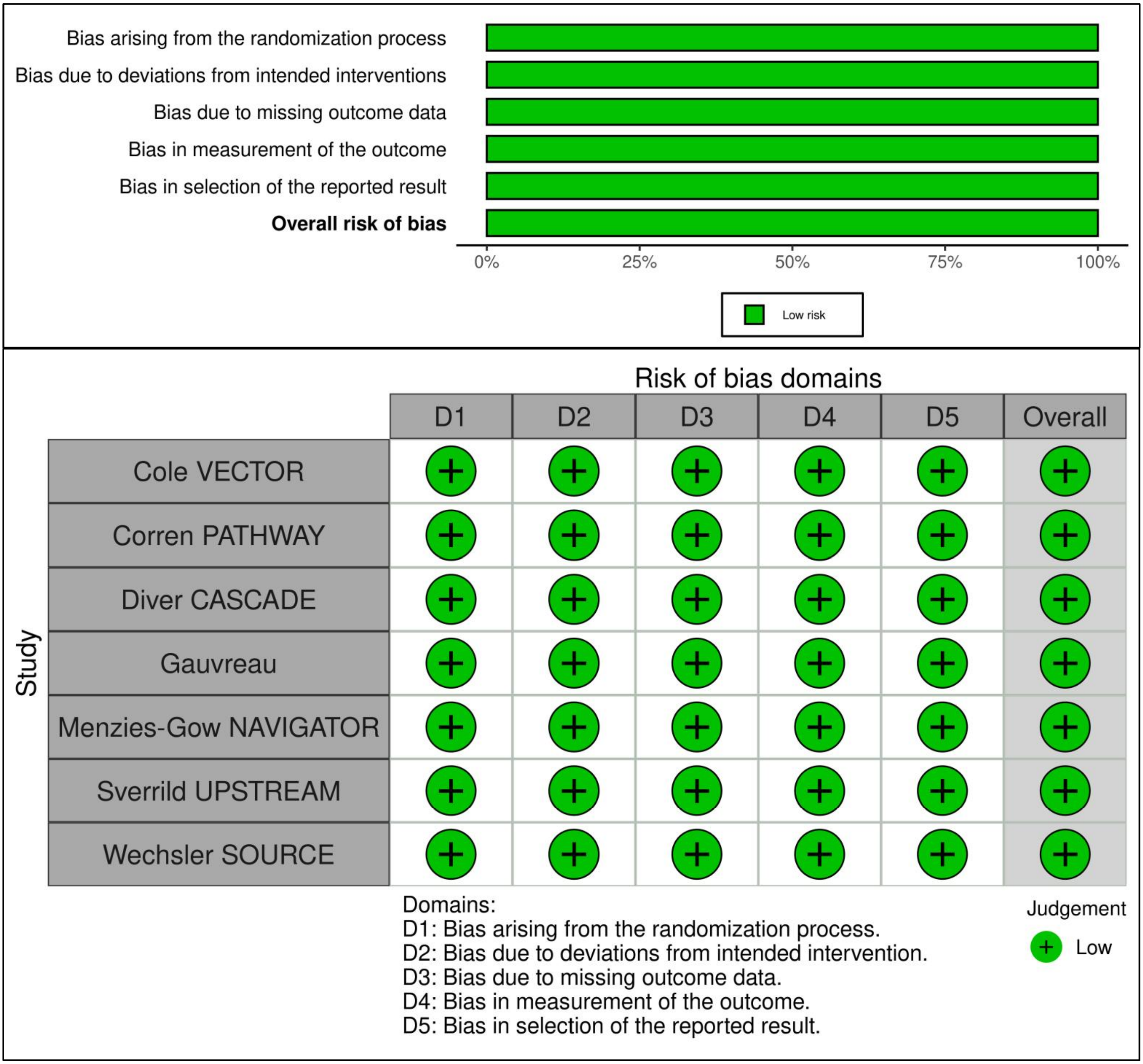
Bias arising from the randomization process
Bias due to deviations from intended interventions
Bias due to missing outcome data
Bias in measurement of the outcome
Bias in selection of the reported result
Overall risk of bias
0%
25%
50%
75%
100%
Low risk
Risk of bias domains
D1
D2
D3
D4
D5
Overall
Study
Cole VECTOR
Corren PATHWAY
Diver CASCADE
Gauvreau
Menzies-Gow NAVIGATOR
Sverrild UPSTREAM
Wechsler SOURCE
Domains:
D1: Bias arising from the randomization process.
D2: Bias due to deviations from intended intervention.
D3: Bias due to missing outcome data.
D4: Bias in measurement of the outcome.
D5: Bias in selection of the reported result.
Judgement
Low
